# Hetero-Pathogenic O181:H4 EAHEC Strain of Sequence Type ST678 Associated with Hemolytic–Uremic Syndrome in Schoolchildren in Russia

**DOI:** 10.3390/microorganisms11071771

**Published:** 2023-07-07

**Authors:** Nikolay N. Kartsev, Elena V. Detusheva, Olga V. Kalmantaeva, Olga V. Korobova, Vladimir N. Gerasimov, Tatiana I. Kombarova, Aleksander I. Borzilov, Nadezhda K. Fursova, Anatoly N. Vereshchagin, Edward A. Svetoch

**Affiliations:** 1 Department of Molecular Microbiology, State Research Center for Applied Microbiology and Biotechnology, Territory “Kvartal A”, 142279 Obolensk, Russia; klub@bk.ru (E.V.D.); n-fursova@yandex.ru (N.K.F.); svetoch@obolensk.org (E.A.S.); 2 Laboratory of Molecular Biology, State Research Center for Applied Microbiology and Biotechnology, Territory “Kvartal A”, 142279 Obolensk, Russia; kalmantaevaov@yandex.ru; 3 Laboratory of Biological Trials, State Research Center for Applied Microbiology and Biotechnology, Territory “Kvartal A”, 142279 Obolensk, Russia; o.v.korobova@yandex.ru (O.V.K.); kombarova.tatyana@yandex.ru (T.I.K.); borzilov@obolensk.org (A.I.B.); 4 Department of Disinfectology, State Research Center for Applied Microbiology and Biotechnology, Territory “Kvartal A”, 142279 Obolensk, Russia; ilcvngerasimov@obolensk.org; 5 N. D. Zelinsky Institute of Organic Chemistry, Leninsky pr. 47, 119991 Moscow, Russia

**Keywords:** EAHEC, hemolytic–uremic syndrome, biofilm, antimicrobials, disinfectants

## Abstract

Background: In the last decade, the importance of hetero-pathogenic enteroaggregative Shiga-toxin-producing *E. coli* for public health has increased. Recently, we described the genetic background of the EAHEC O181:H4 strain of ST678 carrying the *stx2* gene in prophage and five plasmids, including the plasmid-carrying *aggR* and *aaiC* genes. Here, we present the morphological and enzymatic characteristics of this strain, as well as susceptibility to antimicrobials, biofilm formation, etc. Methods: Bacterial morphology was studied using an electron microscope. Susceptibility to antimicrobials was determined using the microdilution method. Cytotoxicity was estimated in Vero cells. Virulence was studied on mice. Results: The morphological and enzymatic properties of the hetero-pathogenic EAHEC strain were typical for *E. coli*; electron microscopy revealed the specific flagella. The strain was susceptible to most antibiotics and disinfectants but resistant to ampicillin and ciprofloxacin and showed a high degree of biofilm formation. Cytotoxicity towards Vero cells was estimated as 80%. Conclusions: The emergence of a new O181:H4 EAHEC strain poses a potential threat to humans because of the virulence potential that must be taken into account in the epidemiological analysis of outbreaks and sporadic cases of foodborne infections associated with hemolytic–uremic syndrome.

## 1. Introduction

Pathogenic *Escherichia coli*, taking into account its localization in the macroorganism and the nature of the pathological processes caused by it, was divided into diarrheal (DEC) and extraintestinal (ExPEC) groups. The DEC group, in turn, contains seven main pathological types (pathotypes): enteropathogenic (EPEC), enterotoxigenic (ETEC), enteroinvasive (EIEC), enteroaggregative (EAEC), enterohemorrhagic (EHEC), diffuse-adhesive (DAEC), and adhesive-invasive (AIEC) [1,2,3]. Each of the listed DEC pathotypes, with the exception of DAEC and AIEC, was identified based on the presence of strictly specific virulence genes in their genomes, determining their pathogenic potential, features of pathogenesis, and symptoms of diseases caused by them. The specific virulence genes were not found in the DAEC and AIEC pathotypes. DAEC was identified based on the presence of afimbrial adhesion genes (*dra* or *afa*), both of which were found also in other groups of pathogenic *E. coli* and in commensals [2]. Moreover, some virulence genes common to the ExPEC were detected in the AIEC strains associated with Crohn’s disease [4].

Accurate identification of the agent pathotype during the DEC and ExPEC infections is one of the key points for choosing effective therapy. However, many studies in the last decades have described strains of *E. coli* that carry virulence factors of more than one pathotype simultaneously; such strains are referred to as hybrid or hetero-pathogenic [1]. For example, the *E. coli* strain of O111:H2 serotype, carrying the Shiga toxin 2 (*stx2*) gene and showing aggregative adhesion (AA) to HEp-2 cells, caused the outbreak of hemorrhagic colitis (HC) and hemolytic–uremic syndrome (HUS) in France in 1992 and was estimated to be a hetero-pathogenic enteroaggregative hemorrhagic *E. coli* (EAHEC) strain [5]. Another hetero-pathogenic EAEC/STEC (enteroaggregative/Shiga-toxin-producing *E. coli*) strain, belonging to the O104:H4 serotype, was identified during the outbreak of foodborne infection that occurred in Germany in 2011 [6,7,8,9,10]. Interest in the hetero-pathogenic *E. coli* pathotypes greatly increased after the last outbreak because 3816 people were involved, including 845 persons who developed HUS and 54 who died [7]. It was shown that increased adhesiveness of the pathogen contributed to greater adsorption of Shiga toxin Stx2 into the bloodstream [11].

The data accumulated to date on the study of *E. coli* strains containing various combinations of virulence genes allowed researchers to identify the following hetero-pathogenic pathotypes among them: STEC/ETEC [12,13,14,15,16,17], EPEC/STEC [18,19,20], EAEC/STEC [5,11,21,22,23,24,25], and EPEC/ETEC [26] as well as a group of hybrid strains carrying combinations of virulence genes ExPEC and DEC [27,28,29,30]. Additionally to hetero-pathogenic EAHEC strains of O111:H2 and O104:H4 serotypes, the EAHEC strains of other serotypes were identified: O86:H_NM_ in Japan [31], O111:H21 in Northern Ireland [23], O59:H- and Orough:H- in Germany [21], and O127:H4 in Italy [32].

The O181 serogroup of *E. coli* was described and included in the *E. coli* serotyping scheme in 2004. The first strain was isolated from beef and was attributed to the O181:H49 serotype; it demonstrated the typical *E. coli* cultural and biochemical properties and carried the *stx2* gene [33]. The strains of the O181 serogroup were also isolated from the patients with diarrhea and HC, meat products, wastewater of cattle farms, and environmental facilities in Finland, Austria, Brazil, Denmark, Vietnam, Germany, Spain, Belgium, Poland, etc. Numerous serotypes for the O181 serogroup were identified: O181:H3, O181:H4, O181:H8, O181:H16, O181:H20, O181:H28, O181:H30, O181:H36, O181:H49, and O181:H-. The strains of listed serotypes were associated with two pathotypes: STEC (O181:H49, O181:H20, O181:H4, O181:H8, O181:H16, and O181:H28) and EAEC (O181:H-, O181:H30, O181:H36, and O181:H3) [33,34,35,36,37,38]. The hetero-pathogenic *E. coli* strain attributed to the EAHEC pathotype of O181:H4 serotype was first presented in our study in 2022 [39].

The purpose of this report is to characterize the phenotypic properties of the EAHEC strain of O181:H4 serotype, which caused an outbreak of HUS among schoolchildren in St. Petersburg, Russian Federation, in 2018.

## 2. Materials and Methods

### 2.1. Ethics Statement

The strain names presented in the study did not contain any personal information such as patient name, age, religion, sex/gender, sexual orientation, etc. In accordance with the regulations of the Russian Federation, every patient entering the hospital signed informed consent to medical procedures and diagnostic tests.

### 2.2. Strains

The *E. coli* strain SCPM-O-B-9427 of the O181:H4 serotype was collected from the patient’s rectal swab received from the Center of Hygiene and Epidemiology in St. Petersburg and deposited into the State Collection of Pathogenic Microbes of the State Research Center for Applied Microbiology and Biotechnology, Obolensk. In silico multi-locus sequence typing (MLST) based on seven loci of house-keeping genes by the Achtman’s MLST scheme database (*adk*_6, *fumC*_6, *gyrB*_5, *icd*_136, *mdh*_9, *purA*_7, *recA*_7) showed the strain SCPM-O-B-9427 belonging to ST678 [39]. The study also used the *E. coli* strain SCPM-O-B-6860 of O104:H4 serotype isolated during the foodborne outbreak with HUS in Germany in 2011, as well as the *E. coli* strains used as control: ATCC 25922 and K12 (C600). The cultures were grown on nutrient agar No. 1 GRM, Tryptone soya agar, Endo agar (SRCAMB, Obolensk, Russia), and MacConkey agar with sorbitol (HiMedia, Maharashtra, India) under aerobic conditions at 37 °C. Bacterial isolates were stored in 20% glycerol at minus 70 °C.

### 2.3. Electron Microscopy

Suspensions of *E. coli* SCPM-O-B-9427 cells were inactivated and fixed in a 4% solution of glutaraldehyde on a 0.1 M cacodilate buffer (pH 7.2) for 24 h at 8 °C. To visualize whole bacteria in a transmission electron microscope, a microbial suspension was applied to copper meshes coated with a formvar film and contrasted with a 1% aqueous solution of uranyl acetate. The prepared samples of the microbial suspension were viewed in an electron microscope Tecnai G2 Spirit BioTWIN (FEI, Holland, Czech Republic) at an accelerating voltage of 120 kV and magnification from 5000 to 40,000 times. The electron beam was carried out using a high-contrast wide-angle CCD camera Gatan Orius SC200W 120 kV (Gatan Inc., Pleasanton, CA, USA), as well as a high-resolution CCD camera Gatan Orius SC1000B 200kV (Gatan Inc., Pleasanton, CA, USA). The photographs were processed using the programs Tecnai Imaging and Analysis, Gatan digital micrograph (FEI, Holland, Czech Republic).

### 2.4. Biochemical Properties

Biochemical properties of the *E. coli* strain were carried out on an automatic microbiological analyzer Vitec 2 Compact (bioMérieux, Craponne, France) using GN maps (bioMérieux, Craponne, France). 

### 2.5. Mobility, Hemolytic Activity, and Colicin Production

The mobility test was carried out by seeding the culture with an injection into semi-liquid agar. The hemolytic activity of the isolates was evaluated after growing them overnight on nutrient agar with 5% of sheep erythrocytes (EcoLab, Elektrogorsk, Russia). The colicinogenicity of isolates was evaluated by the method of delayed antagonism according to Fredericq [40]. Briefly, the studied strain was injected into nutrient agar in Petri dishes and incubated at 37 °C for 18 h. The grown cultures were treated with chloroform vapors for 50 min. Then, 5 mL of semi-liquid (0.7%) nutrient agar, melted and cooled to 45 °C, was evenly distributed over the surface of the agar, mixed with 0.3 mL of a 6-h broth indicator culture of *E. coli* K12. After 18–24 h of incubation at 37 °C, the results were accounted.

### 2.6. Biofilm Formation and Susceptibility to Antimicrobials

The efficiency of bacterial biofilm formation was determined using a method based on the ability of the crystalline violet dye to bind to cells and the biofilm matrix [41]. To obtain biofilms, 96-well flat-bottomed culture plates were used, in which 200 µL of daily bacterial culture was sown at a concentration of 10^6^ CFU/mL and cultured for 24 h at 37 °C. Then, a medium with plankton cells was carefully discarded from the wells and washed for 2–3 min with a sterile PBS buffer (PanEco, Moscow, Russia). Then, 200 mL of filtered 0.1% solution of gentian violet was added into each well, biofilms with dye were incubated for 10–15 min at room temperature, and the dye was completely removed from the wells. The unbound dye was thoroughly washed off with a PBS buffer and dried. After the surface was completely dried, 200 mL of ethanol–isopropanol mixture (1:1) was added to the wells and placed into the novel plates. The optical density of the resulting solution was measured at a wavelength of 590 nm using the XMark Microplate Absorption Spectrophotometer (Bio-Rad, Hercules, CA, USA). The measurement results were interpreted by comparing the OD_590_ values with those of the negative control (pure solvent without the addition of dye). The degree of biofilm production was evaluated in accordance with the recommendations of Rodrigues et al., 2010 [42]. All experiments were carried out in three repetitions.

The minimum inhibitory concentrations (MICs) of 8 antimicrobials of 5 functional groups (amoxicillin–clavulanic acid, cefoperazone–sulbactam, aztreonam, meropenem, amikacin, ciprofloxacin, fosfomycin, and trimethoprim–sulfamethoxazole) were determined according to CLSI recommendations [43] in 96-well flat-bottomed cultural plates. The interpretation of the results was carried out in accordance with the recommendations of EUCAST Version 12.0 (http://www.eucast.org/fileadmin/src/media/PDFs/EUCAST_files/Breakpoint_tables, accessed on 7 March 2022) [44]. 

The minimum bactericidal concentrations (MBCs) of 8 disinfectant compositions of different classes: Dezin (Dezindustria LLC, Moscow, Russia), Triosept-Oxy (NPO SpetsSintez LLC, Moscow, Russia), Neobak-Oxy (NPO Novodez JSC, Moscow, Russia), SAT-22 (Satellit LLC, Moscow, Russia), Biodez-Extra DVU (Biodez LLC, Moscow, Russia), Microbak Forte (BODE Chemie GmbH, Hamburg, Germany), Biodez-Optima (LLC Biodez, Moscow, Russia), and Tristel-Fuse (Tristel Solutions Limited, Snailwell, UK) were determined. The obtained values were presented as a percentage, in accordance with the manufacturer’s instructions [45].

### 2.7. Detection of Shiga Toxin 2 Production

The production of Shiga toxin 2 was carried out according to the method of Parma et al. [46]. Briefly, a suspension of overnight bacterial cultures (optical density of 0.5 U according to McFarland) was prepared and added into 5 mL of Luria Bertani broth (HiMedia, Maharashtra, India) in a ratio of 1:100 and cultured for 6 h at 37 °C with aeration and a rotation speed of 150 rpm. Mitomycin C (PanReac, Barselona, Spain) (0.5 mg/mL) was used for the induction of Shiga toxin 2 production and incubated for 18 h at 37 °C on a rocking chair with a rotation speed of 150 rpm. Cultures were centrifugated at 6000× *g* for 10 min at 4 °C; supernatant was filtered through a 28 mm syringe filter of 0.20 microns (Corning, New York, NY, USA). The qualitative immunoassay was performed using Abraxis Shiga toxin 2 ELISA kit (Abraxis LLC, Warminster, PA, USA) according to the manual of the manufacturer. *E. coli* K12 (C600) strain was used as a negative control and *E. coli* SCPM-O-B-6860 O104:H4 strain as a positive control.

### 2.8. Vero Cell Assay

Cytotoxicity of the culture supernatant of the *E. coli* strain was tested on the Vero cell line ATCC^®^ CCL-81™ (SRCAMB, Obolensk, Russia). The Shiga-toxin-producing strain of *E. coli* SCPM-O-B−6860 was used as a positive control, and the *E. coli* K12 (C600) supernatant was used as a negative control. The toxicity of the supernatants against Vero cells was determined as described previously [47,48,49] with modifications. Briefly, Vero cells were grown in wells of a 96-well culture plate in the amount of 1 × 10^4^ cells/well in a DMEM nutrient medium (Gibco, Hertford, UK) with the addition of 10% fetal bovine serum (HyClone, Traun, Austria) and GlutaMAX-I (Gibco, Hertford, UK) in the volume of 100 µL of full growth medium per well. The plate was placed for 24 h in a CO_2_ incubator (Lamsystems, Miass, Russia) with 5% CO_2_ content at 37 °C to attach cells to the surface of the wells. At the beginning of the experiment, a confluence of about 80% was achieved. The supernatants of the studied *E. coli* strains were added to the experimental wells in fourfold repetitions with a twofold dilution step, and the plate was incubated for 72 h at 37 °C in a CO_2_ incubator at 5% CO_2_ content. Intact wells were used as a negative control (their result was taken as 100% viability of Vero cells); wells with the addition of sodium merthyolate to a final concentration of 0.024% (viability of Vero cells 0%) were used as a positive control. Cytotoxic effect was determined using MTT reagent (PanEco, Moscow, Russia) [50]. For this, 10 mL of MTT stock solution (5 mg/mL) was added to all wells and incubated for 4 h. Then, 150 µL of dimethyl sulfoxide was added into the wells to dissolve formazane crystals, and OD was measured at a wavelength of 540 nm on an XMark spectrophotometer (Bio-Rad, Hercules, CA, USA).

## 3. Results

### 3.1. Electron Microscopy and Cultural Properties

The strain grows well on various nutrient media: on GRM agar, on Tryptone soya agar, Endo agar, and MacConkey media, forming colonies typical of *E. coli* under aerobic conditions at 37 °C for 24 h of growth on them; on Endo and MacConkey media, colonies are colored red and dark red.

**Figure 1:**
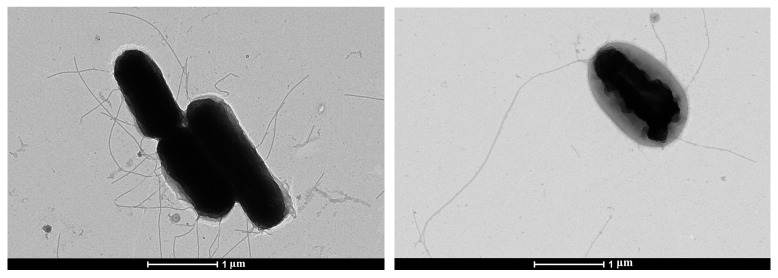
Electron microscopic images of *E. coli* SCPM-O-B-9427.

The study of the morphology of bacterial cells of *E. coli* SCPM-O-B-9427 in a transmission electron microscope by negative contrast showed that the microbial population of bacteria has a relatively homogeneous structure and consists of rod-shaped cells: short sticks, long sticks, and single oval-shaped cells. Bacteria have a width of 0.6 µm to 0.8 µm, and the length of cells is from 1.0 to 2.0 µm. Bacteria in the population have a flat surface and have flagella around the perimeter of the cell (Figure 1).

### 3.2. Biochemical Properties

According to the biochemical properties, the strain *E. coli* SCPM-O-B-9427 was estimated as typical *E. coli* (99%) metabolizing d-glucose, d-maltose, d-mannose, d-sorbitol, l-lactate, sucrose, 5-keto-d-glucanate, d-trehalose, and demonstrating activity of ornithine decarboxylase, lysine decarboxylase, tyrosine amidase, and beta-galactosidase. In contrast with the strain of O104:H4 serotype, the strain of O181:H4 serotype metabolized 5-keto-d-gluconate and alpha-galactosidase but did not metabolize l-lactate and was non-resistant to vibriostatic agent O/129 (Table 1).

### 3.3. Hemolytic and Colicinogenic Activity

The EAHEC strain SCPM-O-B-9427, when growing on nutrient agar with 5% sheep erythrocytes for 24 h, was negative in hemolytic reaction, which was associated with the absence of *hly* genes in its genome. At the same time, the strain produced bacteriocins which inhibit the growth of the indicator strain *E. coli* K12 (C600). The antagonistic phenotype of the strain correlated with its genotype (the *colE* gene encoding colicin E and the *mchB* gene encoding microcin H47) that was presented in our recent publication [39].

### 3.4. Biofilm Formation and Susceptibility to Antimicrobials

It was shown that the EAHEC strain SCPM-O-B-9427 was able to form biofilm at a high level, comparable to the level of biofilm formation of the control strain *E. coli* ATCC 25922. Planktonic cells of both bacterial strains were more sensitive to disinfectants of all used compositions than their biofilms (Table 2).

Interestingly, the MBCs of most disinfectants were higher for the hetero-pathogenic *E. coli* strain SCPM-O-B-9427 than for the control reference strain *E. coli* ATCC 25922, both in planktonic and biofilm forms. This allowed us to assume that the clinical *E. coli* strain was somewhat more tolerant to the tested disinfectants, compared with the reference strain (Table 2).

It was shown that both strains, *E. coli* SCPM-O-B-9427 and *E. coli* ATCC 25922, were sensitive to antibiotics of five functional groups (beta-lactams, aminoglycosides, fluoroquinolones, fosfomycin, and sulfonamides) in planktonic form and in the biofilm state. This is probably due to the fact that amoxicillin–clavulanic acid is able to quickly penetrate into the biofilm structure, destroying it, and also prevents the formation of mature biofilms when using the antibiotic in sub-inhibitory concentrations, as was reported previously [51] (Table 3).

### 3.5. Detection of Shiga Toxin 2 Production

According to the ELISA test, the hetero-pathogenic *E. coli* strain SCPM-O-B-9427 produced Shiga toxin Stx2 in Luria Bertani broth both after mitomycin C induction and without induction.

### 3.6. Cytotoxicity of E. coli Strain SCPM-O-B-9427

**Figure 2:**
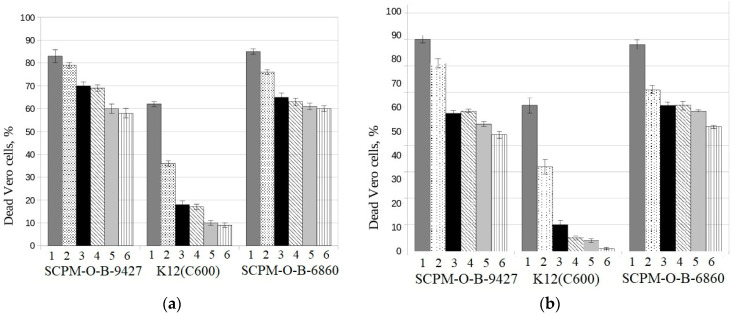
Cytotoxicity of *E. coli* strains SCPM-O-B-9427 of O181:H4 serotype, K12 (C600), and SCPM-O-B-6860 of O104:H4 serotype for Vero cells: (**a**) with mitomycin C induction and (**b**) without mitomycin C induction; 1, 1:2 dilution; 2, 1:4 dilution; 3, 1:8 dilution; 4, 1:16 dilution; 5, 1:32 dilution; 6, 1:64 dilution.

It was shown that the hetero-pathogenic *E. coli* strain SCPM-O-B-9427 of O181:H4 serotype, as well as the control hetero-pathogenic *E. coli* strain SCPM-O-B-6860 of O104:H4 serotype, were toxic for Vero cells: they caused death of more than 80% of Vero cells in 1:2 dilution and 60% of Vero cells in 1:64 dilution. In the same time, the negative control strain *E. coli* K12 (C600), non-toxigenic strain, caused death of 60 and 10% of Vero cells in the same dilutions, both after mitomycin C induction and without induction. The results were statistically significant between cytotoxicity values generated by EAHEC strains and the negative control strain *E. coli* K12 (C600) in all dilutions of tested cultural supernatants (*p* < 0.001) (Figure 2).

## 4. Discussion

Pathogenic *E. coli* continues to pose a serious threat to public health in many developing and developed countries. Every year, more than two million people die from *E. coli* infections in the world. In most cases, the diarrhea disease is caused by the classic pathotypes of *E. coli*, EPEC, ETEC, EAEC, EHEC, and STEC. However, sometimes hetero-pathogenic *E. coli* pathotypes were isolated, in which genomes a combination of virulence genes associated with different DEC or ExPEC strains were detected [1]. Many clinical observations showed that *E. coli* of hetero-pathogenic pathotypes demonstrated a greater pathogenic potential than the strains belonging to classical pathotypes. Such a strain attributed to the hetero-pathogenic EAHEC pathotype, belonging to the O104:H4 serotype, was isolated during a severe outbreak of foodborne infection in Germany in 2011 with numerous cases of HUS and patient deaths [6,7,8,9]. Another example of hetero-pathogenic strains was STEC/ExPEC isolates of the O80:H2 serotype, which carried the combination of Shiga toxin *stx2a* and *stx2b* genes and numerous virulence genes of the ExPEC pathotype and caused not only severe forms of HUS but also posed a threat of bacteremia [52,53]. The hybrid strain of UPEC carrying the *stx* genes has also been described, which caused both diarrhea and urological tract infection (UTI) in the patient [30]. To date, the hetero-pathogenic EAHEC strains of serotypes O111:H2, O104:H4, O86:H_NM_, O111:H21, O59:H-, Orough:H-, and O127:H4 associated with group or sporadic cases of HC and HUS were described [5,21,23,31,32].

The novelty of this paper Is describing the phenotypic properties of a hetero-pathogenic EAHEC strain of O181:H4 serotype that was not previously described for hetero-pathogenic *E. coli* pathotypes. *E. coli* of O181 serogroup were identified in Finland, Austria, Brazil, Denmark, Vietnam, Germany, Spain, Belgium, Poland, Bangladesh, Great Britain, and the USA isolated from the patients with diarrhea and HC, from beef, other meat products, as well as from cattle farm wastewater and environmental facilities [33,34,35,36,37,38]. 

The hetero-pathogenic *E. coli* strain SCPM-O-B-9427 in this study was isolated during outbreak of HUS among schoolchildren in St. Petersburg, Russian Federation, in 2018. This isolate was described as a typical *E. coli* strain by morphological, cultural, and enzymatic properties. In terms of enzymatic activity, this strain was slightly different from the epidemic hetero-pathogenic EAHEC strain SCPM-O-B-6860 collected during a foodborne outbreak in Germany in 2011: in contrast with the strain of O104:H4 serotype, the strain of O181:H4 serotype metabolized 5-keto-d-gluconate and alpha-galactosidase but did not metabolize l-lactate and was non-resistant to vibriostatic agent O/129. Moreover, the *E. coli* strain in our study produced bacteriocins inhibiting the growth of the test strain *E. coli* K12 (C600). The antagonistic phenotype of the strain correlated with its genotype (the *colE* gene encoding colicin E, and the *mchB* gene encoding microcin H47).

It was shown that the *E. coli* strain SCPM-O-B-9427, as well as the control *E. coli* strain ATCC 25922, was characterized by a high level of biofilm formation. Planktonic cells of these strains were more sensitive to disinfectants compared to their biofilms, which was probably due to the presence of extracellular matrix as was reported from other researchers [54,55]. The exception was susceptibility to Tristel-Fuse (consisting of citric acid 5% and activated by sodium chlorite 0.5%), which minimal bactericidal concentrations (MBCs) for planktonic cells were four times higher than for biofilms. Such effect is probably due to the ability of Tristel-Fuse to destroy the biofilm—it was reported previously that chlorine dioxide has a high oxidizing potential and the ability to penetrate through the protective barriers of biofilm, reacting with organic substances on the surface of the cell membrane and disrupting metabolic processes inside the biofilm [56,57]. These data are consistent with the results of the study by Exner et al. on the prevention and control of infections in healthcare institutions, in which it was reported that chlorine dioxide is an effective method of removing and preventing biofilms [58]. When determining the MICs of antibiotics against planktonic cells and biofilms, it was found that both the hetero-pathogenic *E. coli* strain SCPM-O-B-9427 of O181:H4 serotype and the control *E. coli* strain ATCC 25922 were highly sensitive to all tested antibiotics of five functional groups (beta-lactams, aminoglycosides, fluoroquinolones, fosfomycin, and sulfonamides). Of particular interest is the detrimental effect of amoxicillin–clavulanic acid on biofilms: the MICs of antibiotics for *E. coli* biofilms for both strains were 32 times lower than for planktonic cells. This is probably due to the fact that amoxicillin–clavulanic acid is able to quickly penetrate into the biofilm structure, destroying it, and also prevents the formation of mature biofilms when using the antibiotic in sub-inhibitory concentrations, as was reported previously [51].

The hetero-pathogenic *E. coli* strain SCPM-O-B-9427 of O181:H4 serotype was characterized as a producer of Shiga toxin 2 and toxigenic for Vero cells at a level comparable to the reference *E. coli* strain SCPM-O-B-6860.

## 5. Conclusions

A novel hetero-pathogenic enteroaggregative Shiga-toxin-producing *E. coli* strain of the O181:H4 serotype was isolated in the Russian Federation as the agent of HC and HUS foodborne outbreak among schoolchildren in St. Petersburg in 2018. This hetero-pathogenic EAHEC strain was morphologically, culturally, and enzymatically estimated as typical *E. coli*, producing Shiga toxin type 2a, as well as two bacteriocins, colicin E and microcin H47; in addition, it is a high-level biofilm-producer, sensitive to most antibiotics and disinfectants. This strain was toxic for Vero cells.

The emergence of a novel EAHEC strain of O181:H4 serotype is a potential threat to humans that must be taken into account in the epidemiological analysis of outbreaks and sporadic cases of foodborne infections associated with diarrhea, HC, and HUS.

## Figures and Tables

**Table 1:** Biochemical properties of the EAHEC strains of O181:H4 and O104:H4 serotypes.

Test	Description	SCPM-O-B-9427 O181:H4	SCPM-O-B-6860 O104:H4
BGAL	Beta-galactosidase	+	+
dGLU	d-glucose	+	+
OFF	Fermentation of glucose	+	+
dMAL	d-maltose	+	+
dMAN	d-mannitol	+	+
dMNE	d-mannose	+	+
Tyra	Tyrosinarylamidase	+	+
dSOR	d-sorbitol	+	+
SAC	sucrose	+	+
dTRE	d-trehalose	+	+
5KG	5-keto-d-gluconate	+	−
ILATk	l-lactate	−	+
AGAL	Alpha-galactosidase	+	−
ODC	Ornithine decarboxylase	+	+
LDC	Lysine decarboxylase	+	+
CMT	Kumarat	+	+
BGUR	Beta-glucuronidase	+	+
O129R	Resistance to vibriostatic agent O/129	−	+
ELLM	Elman	+	+

Note: +, positive reaction; −, negative reaction; the positions that differ for the strains are highlighted in gray.

**Table 2:** Susceptibility of planktonic cells and biofilms of bacterial strains to disinfectants.

Disinfectant Group	Active Ingredients	MBC, %			
		*E. coli* ATCC 25922		*E. coli* SCPM-O-B-9427	
		P	B	P	B
Dezin	Chlorhexidine bigluconate 20%	0.001	0.02	0.004	0.02
Triosept-Oxy	Hydrogen peroxide 25 ± 7%, didecyldimethylammonium chloride 5 ± 1.5%, auxiliary component—phosphoric acid 1.5%	0.005	0.03	0.03	0.06
Neobak-Oxy	QAS-3%, polyhexamethylene guanidine hydrochloride-3%	0.008	0.06	0.02	0.08
SAT-22	QAS complex 15%, *N*, *N*-bis (3-aminopropyl-dodecylamine) 4%, polyhexamethylene guanidine hydrochloride 5%	0.008	0.1	0.01	0.1
Biodez-Extra DVU	QAS complex 24%, Glutaraldehyde 7%, glyoxal 6%	0.002	0.01	0.008	0.02
Microbak Forte	Benzalkonium chloride 20%, dodecylbispropylenetriamine 5%	0.01	0.04	0.01	0.04
Biodez-Optima	Alkyldimethylbenzylammonium chloride 25%	0.001	0.06	0.004	0.13
Tristel-Fuse	Base: citric acid 5% Activator: sodium chlorite 0.5%	0.5	0.13	1	0.25

Note: P, plankton cells; B, biofilms; QAS, quaternary ammonium salt.

**Table 3:** Susceptibility of planktonic cells and biofilms of bacterial strains to antimicrobials.

Antibiotics	MIC, mg/L (Interpretation)			
	*E. coli* ATCC 25922		*E. coli* B-9427	
	P	B	P	B
Amoxicillin–clavulanic acid	0.008 (S)	<0.0005 (S)	0.008 (S)	<0.0005 (S)
Cefoperazone–sulbactam	<0.0005 (S)	<0.0005 (S)	<0.0005 (S)	<0.0005 (S)
Aztreonam	0.001 (S)	0.13 (S)	<0.0005 (S)	0.13 (S)
Meropenem	<0.0005 (S)	<0.0005 (S)	<0.0005 (S)	<0.0005 (S)
Amikacin	0.002 (S)	0.002 (S)	0.002 (S)	0.002 (S)
Ciprofloxacin	0.001 (S)	0.004 (S)	<0.0005 (S)	0.004 (S)
Fosfomycin	0.01 (S)	0.03 (S)	0.02 (S)	0.03 (S)
Trimethoprim–sulfamethoxazole	<0.0005 (S)	0.002 (S)	<0.0005 (S)	0.002 (S)

Note: P, planktonic cells; B, biofilm; S, sensitive.

## Data Availability

Whole-genome sequence of *E. coli* strain SCPM-O-B-9427 was submitted into GenBank database: CP086259.
